# The water supply system as a potential source of fungal infection in paediatric haematopoietic stem cell units

**DOI:** 10.1186/1471-2334-13-289

**Published:** 2013-06-26

**Authors:** Sabrina Mesquita-Rocha, Patricio C Godoy-Martinez, Sarah S Gonçalves, Milton Daniel Urrutia, Fabianne Carlesse, Adriana Seber, Maria Aparecida Aguiar Silva, Antônio Sérgio Petrilli, Arnaldo L Colombo

**Affiliations:** 1 Departamento de Medicina, Disciplina de Infectologia, Universidade Federal de São Paulo, Rua Napoleão de Barros 590, Vila Clementino, São Paulo 04024-002, Brazil; 2 Instituto de Microbiología Clínica, Edificio de Ciencias Biomédicas, 2 piso, Universidad Austral de Chile, Isla Teja, Valdivia, Chile; 3 Universidad de Antofagasta, Avenida Angamos 201, Antofagasta, Chile; 4 Instituto de Oncologia Pediátrica, GRAACC, Universidade Federal de São Paulo, Rua Botucatu 743, Vila Clementino, São Paulo, SP 04023-062, Brazil

**Keywords:** Filamentous fungi, Nosocomial water, Aspergillosis, Fusariosis, Fungal propagules

## Abstract

**Background:**

We conducted a prospective study to investigate the presence of microfungal contamination in the water supply system of the Oncology Paediatric Institute, São Paulo – Brazil after the occurrence of one invasive *Fusarium solani* infection in a patient after Haematopoietic Stem Cell Transplantation (HSCT). During a twelve-month period, we investigated the water supply system of the HSCT unit by monitoring a total of fourteen different collection sites.

**Methods:**

One litre of water was collected in each location, filtered through a 0.45 μm membrane and cultured on SDA to detect the presence of filamentous fungi. Physicochemical analyses of samples were performed to evaluate the temperature, turbidity, pH, and the concentration of free residual chlorine.

**Results:**

Over the 12 months of the study, 164 samples were collected from the water supply system of the HSCT unit, and 139 of the samples tested positive for filamentous fungi (84.8%), generating a total of 2,362 colonies. *Cladosporium* spp., *Penicillium* spp., *Purpureocillium* spp. and *Aspergillus* spp. were ranked as the most commonly found genera of mould in the collected samples. Of note, *Fusarium solani* complex isolates were obtained from 14 out of the 106 samples that were collected from tap water (mean of 20 CFU/L). There was a positive correlation between the total number of fungal CFU obtained in all cultures and both water turbidity and temperature parameters. Our findings emphasise the need for the establishment of strict measures to limit the exposure of high-risk patients to waterborne fungal propagules.

**Conclusions:**

We were able to isolate a wide variety of filamentous fungi from the water of the HSCT unit where several immunocompromised patients are assisted.

## Background

The expansion of the immunocompromised patient population over the last two decades has led to an increase in the incidence of nosocomial and community-acquired infections by opportunist fungal pathogens, including filamentous fungi. Moulds can enter the hospital environment in many ways; they may survive and proliferate, especially in the presence of moist and unsterile environments. It is well-accepted that the inhalation of airborne fungal propagules is the most relevant route of systemic infection for susceptible patients. Any conditions that enhance the nosocomial dispersion of mould propagules, such as construction, demolition, or dust accumulation during cleaning activities, may increase the exposition of patients to such pathogens [1-3].

Besides the relevance of the airborne fungal propagules for infecting at-risk patients, the presence of *Aspergillus fumigatus* and *Fusarium solani* in the nosocomial water supply has led to speculation that fungal contamination of the nosocomial water supply systems may serve as a route for systemic mould infection. Indeed, it has been demonstrated that fungal propagules may be aerosolised when contaminated water passes through shower heads, taps and toilet bowl, causing respiratory exposure in susceptible patients, especially in areas of major water use, such as showers. Those findings have supported the “wet route” of transmission for human systemic aspergillosis and fusariosis [4-6].

Despite the high incidence of mould infections, especially fusariosis and aspergillosis, in medical centres in Brazil and Latin America, there are few studies available addressing the presence of fungal pathogens in the water supply systems of medical centres in our region [7-9]. The occurrence of *Fusarium* infection in Paediatric Oncology patients undergoing Haematopoietic Stem Cell Transplantation (HSCT) prompted us to investigate the presence of microfungal contamination in the water distribution systems of the Oncology Paediatric Institute – GRAACC – UNIFESP, a tertiary care hospital devoted to the medical assistance of children with cancer.

## Methods

### Setting

**Figure 1:**
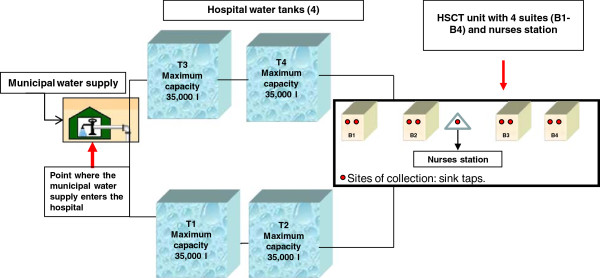
**Water distribution system facilities that were sampled during the environmental surveillance study of pathogenic fungi.** This figure illustrates all collection sites: easel, tanks (TI -T4), sink taps from 4 hospital rooms (B1-B4 represent suites) located on the same floor and a nurses station.

The environmental surveillance of pathogenic fungi was conducted in the Oncology Paediatric Institute of the Federal University of São Paulo (Universidade Federal de São Paulo (UNIFESP), São Paulo, Brazil, a center with 300 new patients/year with a very busy day-hospital and 29 beds, including intensive care and HSCT units. The survey was conducted in the Paediatric Haematopoietic Stem Cell Transplant Recipient unit (HSCT), a division with four bedrooms and a total of six beds, all of which are equipped with a high efficiency particulate air filter (HEPA) and positive pressure (Figure 1).

### Surveys

During a period of twelve months (March 2007-February 2008), we investigated monthly the water system supply of the HSCT unit by monitoring a total of fourteen different collection sites: the location at which the municipal water supply enters the hospital, four cold-water tanks (temperature about 25°C, two aboveground and two underground storage reservoirs) and nine sinks located in four bedrooms: four bathroom taps and one nurses’ station. These four cold-water tanks feeding all units included in this study. Before all water sampling, the target taps were flushed at maximum capacity for 5 minutes to rinse the accumulated dust and dirt from the pipes and tap. Samples from water reservoirs were collected using the Automatic Water Trap (Policontrol, São Paulo, Brazil). Next, each sample bottle was carefully sealed and immediately transported to the laboratory for further analysis. All samples were collected at environmental temperature.

### Physicochemical analysis of water samples

Samples from water taps and tanks were collected every 30–40 days using sterile one-litre glass containers. All samples were collected at environmental temperature. The samples were transported and processed in the Special Laboratory of Mycology (LEMI), Federal University of São Paulo. Physicochemical analysis were systematically performed in triplicate to evaluate i) the concentration of free residual chlorine, using a portable photochlorimeter (poliControl, São Paulo, Brazil); ii) water temperature, using a digital thermometer; iii) turbidity, using a portable turbidimeter (poliControl, São Paulo, Brazil); and iv) pH, using a portable digital device (poliControl, São Paulo, Brazil). All assays were performed according to the manufacturer’s instruction.

### Microbiological analysis of water samples

All laboratory procedures were safely performed under a biological safety cabinet. The water samples (1 litre) were filtered through 0.45 μm Millipore membranes (Millipore, São Paulo, Brazil) using a manifold vacuum filtration system. The membranes were cultured on Sabouraud dextrose Agar plates (SDA) containing chloramphenicol (Oxoid). All plates were incubated at 25°C and 37°C for a maximum period of 15 days and were checked daily for the presence of filamentous fungi. Colony counts were enumerated as colony-forming units (CFU) per litre. The isolated colonies that exhibited different morphological aspects were subcultured and stocked in glass tubes with SDA slants for further identification.

### Morphological identification of filamentous fungi

Subcultures of the stock collection were assayed on three different media to stimulate the development of fruiting bodies: Malt Agar, Potato Agar and Oat Agar plates (Becton Dickinson, Spark, USA). Fungal isolates were identified according to established methods based on macroscopic and microscopic features [10,11].

### Statistical analysis

Statistical analysis of the data was performed using the programme R Development Core Team (2005), Vienna, Austria, which is available at http://www.R-project.org. The correlation between CFU and the physicochemical parameters was determined using the Pearson’s Correlation Coefficient.

## Results

Over the 12 months of the study, 164 samples were collected from the water supply system of the HSCT unit. Of these, 139 tested positive for filamentous fungi (84.8%), generating a total of 2,362 colonies representing eleven different fungal genera. The water from tanks and taps was more contaminated than the samples obtained from the point at which the municipal water supply enters the hospital. Combining all of the 139 positive cultures obtained during the study, we found 36 CFU of fungal isolates growing in the samples from the municipal water supply at the entrance to the hospital, 1,173 CFU in tanks and 1,153 CFU in tap water samples (Table 1).

**Table 1:** Distribution of fungal isolates obtained from the water supply system of a paediatric haematopoietic stem cell unit during a 12-month period

**Sampling sites**	**Number of positive samples/total samples collected (%)**	**Mould recovered**	Total CFU*	**Mean (range) concentration CFU/L**
	3 (27.4)	*Mycelia sterilia*	4	1.3 (1–2)
Point where the municipal water supply enters the hospital:	2 (18.2)	*Penicillium* spp.	10	5 (5–5)
11 samples collected	2 (18.2)	*Cladosporium* spp.	6	3 (2–4)
	1 (9.1)	*A. niger sensu lato*	1	1 (1–1)
	1 (9.1)	*Trichoderma* spp.	15	15 (15–15)
		Subtotal	36	
	33 (70.2)	*Penicillium* spp.	398	12.1 (1–55)
	28 (59.7)	*Cladosporium* spp.	262	9.4 (2–20)
	23 (48.9)	*Mycelia sterilia*	192	8.3(1–25)
Tanks:	10 (21.3)	*A. flavus sensu lato*	55	5.5 (1–20)
47 samples collected	6 (12.8)	*A. niger sensu lato*	38	6.3(1–15)
	5 (10.6)	*Aureobasidium pullulans*	28	5.6 (2–15)
	2 (4.3)	*Fusarium solani* complex	40	20 (20–20)
	2 (4.3)	*Trichoderma* spp.	20	10 (10–10)
	2 (4.3)	*Chrysonilla sitophila*	13	6.5 (3–10)
	2 (4.3)	*Purpureocillium lilacinum*	21	10.5 (1–20)
	2 (4.3)	*Rhizopus* spp.	55	27.5 (5–50)
	1 (2.1)	*A. deflectus sensu lato*	25	25 (25–25)
	1 (2.1)	*A. oryzae sensu lato*	1	1 (1–1)
	1 (2.1)	*Aspergillus* spp.	20	20 (20–20)
	1 (2.1)	*Mucor* spp.	5	5 (5–5)
		Subtotal	1173	
	23 (21.7)	*Purpureocillium lilacinum*	563	24.5 (2–60)
	15 (14.2)	*Cladosporium* spp.	148	9.9 (2–28)
	14 (13.2)	*Fusarium solani* complex	216	15.4 (2–80)
	14 (13.2)	*Mycelia sterilia*	94	6.7 (2–24)
	9 (8.5)	*Penicillium* spp.	60	6.7 (2–24)
Tap water from high-risk unit (HSCT):	6 (5.7)	*A. flavus sensu lato*	18	3 (2–8)
106 samples collected	2 (1.9)	*Aureobasidium pullulans*	44	22 (4–40)
	1 (0.9)	*Aspergillus* spp.	4	4 (4–4)
	1 (0.9)	*A. sydowii sensu lato*	2	2 (2–2)
	1 (0.9)	*Fusarium dimerum* complex	4	4 (4–4)
		Subtotal	1153	
		**TOTAL**	**2362**	

*Total number of CFUs obtained in all positive samples.

The prevalence of fungal genera and quantity of fungal colonies exhibited considerable variation between the different compartments of the water supply system evaluated during our study. As summarised in Table 1, besides unidentified moulds (*Mycelia sterilia*), *Cladosporium* spp., *Penicillium* spp., *Purpureocillium* spp. and *Aspergillus* spp. were ranked as the most frequently isolated genera of moulds in all of the collected samples.

*Purpureocillium lilacinum* was mainly isolated from samples of tap water; of the 106 cultures obtained from tap water (range of 2–60 CFU/L, total of 563 CFU), 23 (21.7%) were positive for this species. *Penicillium* spp. were isolated from 70.2% of all cultures collected from tanks (range of 1–55 CFU/L), 2 out of 11 (18.2%) samples from the municipal water supply at the entrance to the hospital and 9 out of 106 (8.5%) samples from tap water (range of 2–24 CFU/L).

*Cladosporium* spp. were isolated from 28 out of 47 (59.7%) samples obtained from tanks (range of 2–20 CFU/L), 2 out of 11 (18.2%) samples from the point where the municipal water supply enters the hospital (range of 2–4 CFU/L) and 15 out of 106 (14.2%) cultures from tap water (range of 2–28 CFU/L).

*Aspergillus* species were mostly observed by the isolation of *A. flavus sensu lato* and *A. niger sensu lato*. *Aspergillus flavus sensu lato* was isolated from 10 out of 47 (21.3%) samples from tanks (range of 1–20 CFU/L) and 6 out of 106 (5.7%) samples from tap water (range of 2–8 CFU/L). *Aspergillus niger* was isolated from 1 out of 11 (9.1%) samples from the point where the municipal water supply enters the hospital (range of 15 CFU/L) and 6 out of 47 (12.8%) samples from tanks (range of 1–15 CFU/L). We were not able to isolate any cultures of species from the A*spergillus* section *Fumigati*.

Notably, *Fusarium solani* complex isolates were obtained from 2 out of 47 samples collected from tanks and 14 out of 106 samples collected from tap water, exhibiting a mean concentration of propagules ranging between 15.4 and 20 CFU/L, respectively. In addition, we found 1 out of 106 samples collected from tap water to be positive for *Fusarium dimerum* complex (4 CFU/L).

**Figure 2:**
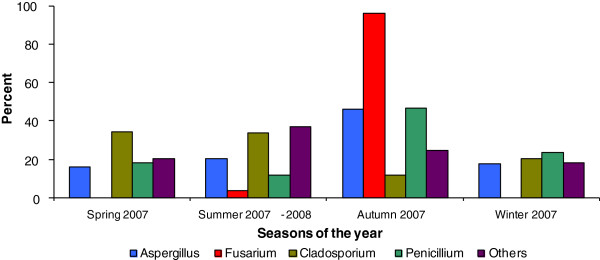
Distribution of fungal propagules in water samples collected from 4 different seasons of the year.

Figure 2 illustrates the distribution of filamentous fungi counts obtained in each of the four different seasons. We observe *Aspergillus* spp. was isolated from water sample cultures in all of the different seasons, and *Fusarium* spp. was isolated only in the autumn and summer collections. Overall, a larger number of fungal isolates was obtained from cultures collected during autumn and summer months than in the winter or spring.

**Figure 3:**
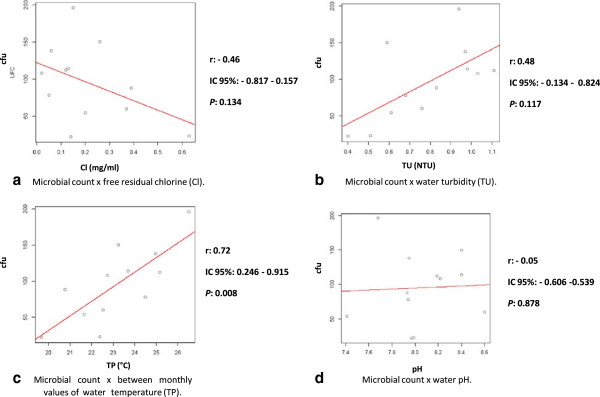
**a-d.** Correlation between microbial counts and four different the physicochemical parameters of water samples documented during a 12-month period of study.

The Figure 3a-d illustrates the relationship between four different physicochemical parameters and the counts of water samples collected over a 12-month period. There was a positive correlation between the total number of fungal CFUs obtained in all cultures and both water turbidity and temperature parameters. However, the correlation between these variables only achieved statistical significance in the comparison of microbial counts and water temperature (r = 0.72 and *p* = 0.008). Finally, there was a trend towards inverse correlation between fungal counts and residual chlorine, but without statistical significance (r = 0.46 and *p* = 0.134).

Despite all positive cultures from the water supply, none of the 25 children transplanted within the study period, 13 autologous and 12 allogeneic transplants have had any invasive fungal infection. Indeed, the unit was renewed before this study to remove the toilets’ water reservoirs and the showers heads. All patients since them have dry baths or use mineral water to clean. No other infections were observed after these measures.

## Discussion

Studies conducted worldwide have shown that filamentous fungi may be isolated from nosocomial water supply systems. However, there is no standard method for processing cultures that are obtained from samples representative of the water distribution system. Indeed, publications on this topic use different sampling methods, different volumes, and a large diversity of culture media and processing techniques to concentrate the inoculum to be identified [3,12-15]. Consequently, in the absence of a standard method for recovering fungi from water samples, we decided to optimise our processing by sampling large volumes of water (1 L) from different sites and using a membrane filtration methodology to concentrate the inoculum to be cultured. We have chosen a culture medium that is non-selective for fungi (Sabouraud Dextrose Agar) to support the growth of a greater number of microorganisms.

The lack of a consensus definition of the best methodology to recover fungi from water systems also limits the ability to compare the results generated by different investigators. It is reasonable to expect that the concentration of fungal propagules may differ considerably depending on the methods used for collecting and processing the samples. In addition, it is also possible that regional characteristics of climate and biomes may also influence the composition of anemophilous fungal isolates eventually found in water supply systems [4,5,13,15-20].

Our study was carried out over a one-year period in a HSCT unit, where preventive measures to reduce patients’ exposure to fungal nosocomial propagules have always been used, including the use of a HEPA air filtration system, positive pressure in the bedrooms and sealing off the windows. All patients use N-95 masks when they are outside their room. After a previous case of *Fusarium* infection, which prompted renovations including removal of the water reservoir in the toilets and this study, the only change in the patients care was restriction on showering [21].

Apart from *Mycelia sterilia,* we were able to identify eleven different genera of filamentous fungi in the following prevalence: *Cladosporium* spp. >*Penicillium* spp. >*Purpureocillium* spp. >*Aspergillus* spp. This finding is in accordance with other investigations that found a similar prevalence of fungal genera isolated from hospital water systems [3,4,22,23]. Despite all the positive cultures, none of them has caused any infection in the HSCT patients within the study period.

Considering all of the fungal propagules isolated from the nosocomial water system, we found differences in the fungal concentrations obtained from different compartments. *Fusarium solani* complex species exhibit high concentrations in tap water (216 CFU) when compared to the concentrations of these species present in water samples obtained from the tanks (40 CFU). Previous studies have also demonstrated the occurrence of *Fusarium* in hospitals tap water. Indeed, an investigation conducted in a Texas hospital, USA, which was carried out over ten years, demonstrated the colonisation of *Fusarium* species in the hospital hydraulic system; there, the concentrations of *F. solani* were shown to be ≥1,000 CFU/mL [24]. Sautour et al. [25] also reported the occurrence of high concentrations of *Fusarium* spp. in tap water and showers from two French hospitals (>10^5^ CFU/mL). These data suggest that the production of the biofilm inside the hydraulic system may contribute to amplifying the fungal inoculum in the water system.

*Aspergillus flavus sensu lato* and *A. niger sensu lato* were the most commonly discovered isolates of the genera *Aspergillus*. It is important to note that *Aspergillus* propagules were present at higher concentrations in tank water compared to tap water (total of 139 CFU and 24 CFU, respectively). In contrast to previous studies, we were unable to isolate species representative of the *Aspergillus* section *Fumigati* in the water system [22,23,26-28].

Zygomycetes members were isolated in limited concentrations and only from samples taken from tanks. Of interest, a recent study conducted in Belgium found that only Hyphomycetes members were isolated in shower and tap water samples from a tertiary hospital [26]. Results obtained by our group and by Hayette et al. [26] may suggest that those microorganisms are difficult to isolate in culture or they may be unable to grow and survive in the hospital water supply system. However, considering that other investigations have isolated *Mucor*, *Rhizopus* and *Absidia* from water samples, it is more reasonable to suppose that the media and culture conditions utilised in this study were not appropriate for the isolation of Zygomycetes [5,22,27].

In our study, the highest concentrations of fungi in the water system were found in autumn and summer seasons. Other investigations which have included water microbiological monitoring from hospitals have not found large differences in the fungal isolates between different seasons [3,23].

We evaluated the correlation between the physicochemical parameters and the total concentration of fungal propagules in the samples collected. In the present study, water temperature was the only physicochemical parameter that exhibited a strict correlation with the number of fungal propagules isolated from all sites cultured. We were surprised that the residual chlorine did not impact the total number of propagules obtained in our sampling (*p* = 0.134). Of note, throughout the study period, the free residual chorine rate varied from 0.14-0.89 mg/mL, with a mean of 0.38 mg/mL. These values are consistent with those established by the Brazilian Ministry of Health, ordinance nº 518/2004, which set the standard for drinking water in Brazil during the period of the study [29]. Nagy & Olson [19], in a study conducted in California, USA, monitored the municipal water distribution systems and concluded that the treatment of potable water with chlorine does not inhibit the development of fungi. Other researchers have documented that the mould conidia may be more resistant to chlorine [30].

## Conclusions

In conclusion, we were able to isolate a wide variety of filamentous fungi from the water of the HSCT unit where several immunocompromised patients are assisted. None of these isolates have caused any invasive infection. These findings emphasise the need for the establishment of strict measures to limit the exposure of high-risk patients to waterborne fungal propagules. The use of dry baths or mineral water is exceedingly cheap and may have an important role to protect the patients from invasive infections.

This work was developed in the Laboratório Especial de Micologia, Escola Paulista de Medicina - Universidade Federal de São Paulo - approved by Institutional Review Board (trial 0541/07).

## Competing interest

The authors declare that they have no competing interests.

## Authors’ contributions

SMR contributed in design and managed the study, processed all samples, conducted the analysis and drafted the paper. PCGM designed and managed the study, performed all samples, conducted the analysis the paper. SSG contributed in the processing of samples, conducted the analysis and drafted the paper. MDU conducted the statistical analysis. FC, AS, MAS, ASP conducted the clinical analysis the paper, and conducted infection vigilance in period this study and drafted the paper. ALC designed and managed the study, conducted the analysis the paper, and drafted the paper. All authors read and approved the final manuscript.

## Pre-publication history

The pre-publication history for this paper can be accessed here:

http://www.biomedcentral.com/1471-2334/13/289/prepub
